# Detecting Miscoded Diabetes Diagnosis Codes in Electronic Health Records for Quality Improvement: Temporal Deep Learning Approach

**DOI:** 10.2196/22649

**Published:** 2020-12-17

**Authors:** Sina Rashidian, Kayley Abell-Hart, Janos Hajagos, Richard Moffitt, Veena Lingam, Victor Garcia, Chao-Wei Tsai, Fusheng Wang, Xinyu Dong, Siao Sun, Jianyuan Deng, Rajarsi Gupta, Joshua Miller, Joel Saltz, Mary Saltz

**Affiliations:** 1 Department of Computer Science Stony Brook University Stony Brook, NY United States; 2 Department of Biomedical Informatics Renaissance School of Medicine at Stony Brook Stony Brook, NY United States; 3 Department of Applied Mathematics and Statistics Stony Brook University Stony Brook, NY United States; 4 Department of Medicine Renaissance School of Medicine at Stony Brook Stony Brook, NY United States

**Keywords:** electronic health records, diabetes, deep learning

## Abstract

**Background:**

Diabetes affects more than 30 million patients across the United States. With such a large disease burden, even a small error in classification can be significant. Currently billing codes, assigned at the time of a medical encounter, are the “gold standard” reflecting the actual diseases present in an individual, and thus in aggregate reflect disease prevalence in the population. These codes are generated by highly trained coders and by health care providers but are not always accurate.

**Objective:**

This work provides a scalable deep learning methodology to more accurately classify individuals with diabetes across multiple health care systems.

**Methods:**

We leveraged a long short-term memory-dense neural network (LSTM-DNN) model to identify patients with or without diabetes using data from 5 acute care facilities with 187,187 patients and 275,407 encounters, incorporating data elements including laboratory test results, diagnostic/procedure codes, medications, demographic data, and admission information. Furthermore, a blinded physician panel reviewed discordant cases, providing an estimate of the total impact on the population.

**Results:**

When predicting the documented diagnosis of diabetes, our model achieved an 84% F1 score, 96% area under the curve–receiver operating characteristic curve, and 91% average precision on a heterogeneous data set from 5 distinct health facilities. However, in 81% of cases where the model disagreed with the documented phenotype, a blinded physician panel agreed with the model. Taken together, this suggests that 4.3% of our studied population have either missing or improper diabetes diagnosis.

**Conclusions:**

This study demonstrates that deep learning methods can improve clinical phenotyping even when patient data are noisy, sparse, and heterogeneous.

## Introduction

The widespread adoption of an electronic health record (EHR) has generated large amounts of data, providing an opportunity for clinical phenotyping to identify patients with characteristics of interest [1,2]. Analyzing these rich EHR data has many potential uses such as predicting mortality, defining cohorts, evaluating health care policy, and driving health care finance that affect patient care, revenue, and performance evaluation. The ability to use large amounts of clinical data to discover or validate information is of particular interest for research studies as well as clinical practice [3]. Over the years, disease phenotyping methods from EHR data have evolved from traditional manually developed rule-based analysis for concept curation such as eMERGE and PheKB [4-6] to statistical and traditional machine learning techniques [7-9], and more recently, deep learning techniques which offer better performance while reducing the need for data preprocessing and feature engineering [10-12]. However, EHR data are often incomplete, inaccurate, fragmented, and heterogeneously structured, reflecting the challenges of real-world information gathering, extraction, and interpretation [1,4,13].

Being able to accurately predict diseases in a population could lead to targeted clinical interventions [14], while applying predictive models retrospectively may identify patients with incorrect or missing diagnoses, documentation, or billing codes. We chose diabetes mellitus for such phenotyping applications because it is a highly prevalent disease with heterogeneous presentations and objective diagnostic criteria. In the United States, more than 34 million people have diabetes, and 1 out of 4 people are undiagnosed. Diabetes is associated with many serious medical comorbidities such as heart disease and stroke, as well as high costs of medical care [15]. Previous efforts assessing errors in diagnosis, classification, and disease coding in patients with diabetes using clinical trial data and primary care data have shown that significant errors from misdiagnosis, misclassification, and miscoded patient data are associated with worse therapeutic outcomes [16-21].

In this study, we aim to characterize clinical phenotype for diabetes using data available at the time of discharge by using a generalizable sequential-based deep learning method. We employ all laboratory results, medications, demographic data, and other admission data such as days from prior admission or duration of current visit for each patient. We also include diagnostic codes and procedure codes from all encounters except the most recent one, which is the target to predict. The goal of this work is to train a model that can identify diseases—diabetes in this study—for each patient based on all available information. This model has the potential to merge into hospital real-time monitoring systems for flagging patients, potentially improving patient care and EHR documentation quality, among countless other downstream benefits.

In recent years, there are many interesting studies applying deep learning methods on EHR data. Using dense neural networks (DNNs) for finding patients at high risk of mortality [22], discovering characteristic patterns of physiology [23], representing patient data for machine learning purposes [14], improving coding accuracy in EHR data [24,25], taking advantage of recurrent neural networks (RNNs) for predicting future diagnosis codes and clinical events [26-30], forecasting kidney transplant success [31], early detection of heart failure [32], using bidirectional RNNs for medical event detection [33], and combining convolutional neural networks and RNNs for improving patient representation [34] are just a few of these inspiring projects. There are extensive survey papers exploring and categorizing recent projects based on methods and their goal [35,36]. However, in most of them limited EHR data elements are used, patients have extensive background information, and the goal is to predict what is recorded in a future visit for a patient. The real-world disease classification problem in a health system is different and requires a more general and scalable model that can make robust predictions using all data elements.

Our study offers the following key contributions: (1) A minimally curated, real-world data set for model training is employed, where about 76% of patients had only 1 encounter, reflecting the incomplete and fragmented nature of EHR data. (2) Data from 5 different health care facilities in the United States are combined to show the generalizability of the model, avoiding overfitting on a single facility, and demonstrating the capability of neural networks to learn from data with diverse and complex structures. (3) Precise measurements are provided to show improvements and performance of this model. (4) A thorough validation with a panel of clinicians is conducted to adjudicate the clinical phenotype from longitudinal data in cases where the model disagreed with the documented disease coding. (5) The total impact on the population for patients is calculated with both improper and missed diagnosis codes in their EHR data.

## Methods

### Data Set Description

We obtained data from the CERNER Health Facts database, a large multi-institutional deidentified database derived from EHR data and administrative systems. The database has 599 facilities. For this study, we extracted inpatient encounter data from the 5 acute care facilities with the most inpatient discharges from January 1, 2016, to December 31, 2017. The extracted encounters all have ICD-10 (International Classification of Diseases, 10th edition) diagnosis codes and at least one laboratory test. Table 1 summarizes general information including statistics on the reported cases of diabetes in each facility and the mean number of medications and unique laboratory tests. Population demographic information is summarized in Multimedia Appendix 1.

**Table 1:** General and diabetes-related inpatient statistics in facilities studied.

Facility ID	131	143	384	898	1157
Number of encounters	62,318	60,175	45,390^a^	55,444	52,080
Number of patients	41,854	38,657	31,387^a^	38,953	36,336
Mean number of ICD^b^ codes	13.74	19.07	3.61^a^	13.77	10.22
Percentage of encounters with diabetes	34.55	27.82	9.93^a^	23.34	25.91
Mean number of medications	21.56	12.58	16.43	1.71^a^	8.79
Percentage with metformin	3.06	0.76	1.53	0.08^a^	0.28
Mean number of unique laboratories	56.72	49.94	26.89^a^	48.73	61.7
Percentage with hemoglobin A1c (HbA1c)	28.91	13.20	0.00^a^	24.16	19.47

^a^ICD: International Classification of Diseases.

^b^The lowest value in each row.

EHRs from different facilities usually have various formats, structures, and may not be directly interoperable. For this reason, demographic information, laboratory results, diagnosis codes, procedure codes, and medications were mapped to the Observational Health Data Sciences and Informatics (OHDSI) Common Data Model (version 5.3; vocabulary release on October 2, 2018), a standard data model for observational health studies [37-39]. Clinical notes are not available in the database and were not included in this study.

#### Laboratory Tests

There are 2 major challenges for representing laboratory values. First, laboratory tests may be performed multiple times in a single encounter. Second, there are a large number of test types, which form a huge sparse matrix with many missing values. We proposed 2 approaches to represent laboratory tests: (1) We used statistical summaries including median, max, min, total count, and the values of the first and last instance of a laboratory test for each single encounter. A laboratory test is ordered by a physician if there are concerns that it may not be normal. Therefore, when it is unavailable the value is either expected to be normal or its result is reflected in other available features clearly. For these laboratory tests we used median imputation for filling missing values. It is worth mentioning that we explored more complicated imputation methods as well, including MICE [40], Soft-Impute [41], and SVD-Impute [42]. However, these methods did not provide distinct improvement and took much more computation power. (2) We counted the number of laboratory values that were classified as “high,” “low,” “within the range,” or “normal,” “abnormal,” and “unspecified” according to standards provided by each facility. In a case that a laboratory value is not recorded, these values are exactly 0, thus imputation is not needed. However, ranges for some features are undefined in the EHR system that makes it necessary to have numerical values as well.

#### Diagnosis and Procedure Codes

Because the model is designed to use all information available at the time of discharge, codes from past encounters are included. However, the codes for the current encounter are the target to be predicted and not included in the input feature matrix. Codes are represented as binary values for each ICD code in the data set.

#### Medications

Medications were mapped from National Drug Codes to RxNorm’s Concept Unique Identifiers using mappings associated with the OHDSI-controlled vocabularies. Total counts of drug exposure and per inpatient visit were added to the feature matrix.

#### Demographic/Personal Information

We also included age, weight, height, race, ethnicity, and gender from the data set. For categorical features (race, ethnicity, and gender), we added them to the feature matrix through one-hot encoding.

#### Derived Features

We further derived calculated features, such as the number of days from the latest previous encounter, days hospitalized, and the facility IDs represented with a one-hot encoding scheme.

#### Target

The ICD-10-CM codes that defined clinical diabetes were derived from the Clinical Classification Software (CCS) [43] categories 49, 50, and 186. We excluded conditions that do not clearly fit the clinical definition of diabetes as a chronic disease, such as “unspecified hyperglycemia,” “prediabetes,” and “gestational diabetes.” All ICD codes under the mentioned CCS codes were included except conditions specified in Multimedia Appendix 2.

In order to reduce the sparsity of the feature matrix and remove features that are not available or relevant to the target disease, we only kept features with a nonzero value and appearing in at least 5% of positive cases in the training set.

### Data Vectorization

**Figure 1:**
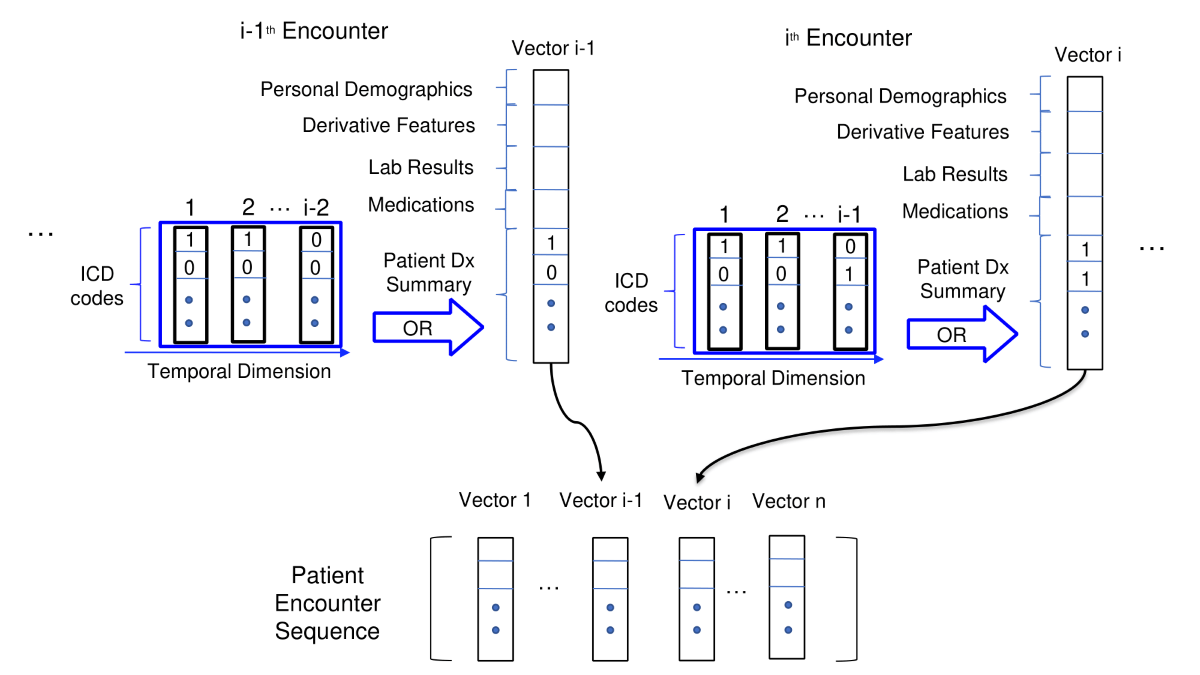
Feature matrix construction from patient encounters. All information from the ith encounter, except ICD codes, was combined with ICD codes from prior encounters to build a slice in the sequence. Dx: diagnosis code; ICD: International Classification of Diseases.

As previously mentioned, diagnosis and procedure codes from the final encounter are the prediction goal and are not included in the input to the model. We combined the target diagnosis codes using CCS categorization to create a binary value for the presence of disease. For each encounter i, we created a vector v_i_ by concatenating laboratories, medications, demographics from the *i*th encounter, and the accumulated ICD code presence value from prior encounters: “0” for no presence and “1” for at least one instance as shown in Figure 1. The idea behind this “or” operation is to represent the history data as physicians would review them, that is, focusing on the presence or absence of diseases in the patient history. Thus, in mimicking our stated goal, these vectors hold the information that would be available at the time of discharge when the codes must be determined.

### Machine Learning/Deep Learning–Based Predictive Models

We employed both nonsequential and sequential models in this study. In nonsequential models, the order of input features does not matter and does not distinguish features based on their temporal occurrence. On the contrary, sequential models care about which features happened when and they are designed to capture temporal information.

#### Nonsequential Models

We took 2 traditional machine learning approaches, random forest and logistic regression, as baselines for comparison. Furthermore, we took advantage of DNNs which are powerful classifiers and have been widely used in previous studies [22-25]. The main advantage of DNNs over other machine learning methods is the capability to learn patterns more effectively from large data sets with numerous features without the need for feature selection.

#### Sequential Models

Because of the inherently sequential nature of a patient’s medical history, we expect that sequential models should outperform those that do not consider the order of inputs. RNNs are among the most powerful tools for prediction and classification when there is a sequence of data leading to the result. Standard or vanilla RNNs face vanishing and exploding gradients in back-propagation during the training phase as the longer the sequence of inputs grows, the longer and more unstable the chain of gradients becomes to calculate. Because of these problems, we leveraged long short-term memory (LSTM) [44] and gated recurrent unit (GRU) [45] which use “forget” and “update” elements to selectively turn off portions of the model, effectively reducing the parameter space during each training step. Furthermore, we added additional dense layers after the output of recurrent layers [46,47]. We call these models LSTM-DNN and GRU-DNN, respectively.

### Model Training

As is the case in almost any phenotyping study, the data set is imbalanced, with only 21.59% of cases positive for diabetes. In this subsection, we briefly go through techniques and parameters used to increase prediction power and avoid overfitting. These parameters also make it possible to replicate experiments. Data set is normalized (mean = 0, variance = 1) before training to improve performance and stability. The data set (combination data of 5 acute care facilities that were mentioned earlier) was split using stratified random sampling to 80% for the training set and 20% for the test set. The training and test sets were the same for training and evaluation of all models.

#### Traditional Machine Learning Methods

For the logistic regression model, we used L2 regularization (1.0) and in the random forest model we limited the tree maximum depth to 30. The class weights for both models were adjusted inversely proportional to class frequencies to give more weight to the minor class (positive cases).

#### Neural Networks

For the DNN model both L2 regularization (0.0002) and dropout (with rate 0.45) [48] were used. We applied weight balancing with log proportion as the prevalence ratio (2.22) to calculate loss in each epoch. We employed mini batches (2048) which are more computationally efficient, use less memory, and are generally more robust as they avoid local minima in optimization steps [49]. After hyper-tuning using 12.5% of training data for cross validation, the best model was trained with mean squared error loss, Adam optimizer [50], Xavier uniform initializer [51], tanh activation functions in hidden layers, and a sigmoid activation function in the output layer. The dense network consists of 4 hidden layers (512, 512, 512, 512) and the recurrent networks have 2 recurrent layers (512, 512) (LSTM/GRU) and 2 dense layers (512, 512). All have a single neuron output. Adding additional embedding layers did not improve models’ performances.

As the search space is enormous, we had 2 steps for finding the best parameters. First, we fixed all parameters except one and hyper-tuned that specific parameter. After reaching a short list of candidates for each variable, we used grid search on all of them to find the best combination. The network configuration was reached by extensive hyperparameter search over the following parameters: activation functions (tanh, relu, selu), loss functions (mean squared error, mean absolute error, binary cross entropy), optimizers (Adam, sgd), batch size (512, 1024, 2048), L2 regularization (0.001, 0.01, 0.10, 0.05, 1, 2, 10), dropout rate (from 0 to 0.80 every 0.05), number of layers (1 to 7), and various number of neurons in each layer (different combinations of powers of 2 as expected to be faster while using GPU nodes).

### Review Panel Validation Method

Identifying inaccuracy in coded disease states was a major motivation for the study, and we hypothesized that a well-trained model would be accurate even when some diagnosis codes in the training set were incorrectly coded. Because it is impossible to evaluate this goal using existing diagnosis codes which themselves can be flawed, we asked 3 board-certified practicing physicians to review cases where the model contradicted the documented diagnosis. In this experiment, experts were provided with the same information as the model, including all demographic information, laboratory results, and medications as well as event timelines for inpatient encounters. Furthermore, the experiment was performed in a blinded manner—experts did not have any knowledge of the diagnosis from either the model prediction or EHR documentation. We believe this experiment can shed light into the usefulness of such a model for flagging cases in hospital systems.

## Results

### Experimental Setup

For training and testing the deep learning models, we used Keras framework [52] backed by Tensorflow [53] and the scikit-learn library [54]. The training was performed on a NVIDIA Tesla V100 GPU with 640 Tensor Cores.

### Performance of Phenotyping Diabetes According to EHR Labels

**Figure 2:**
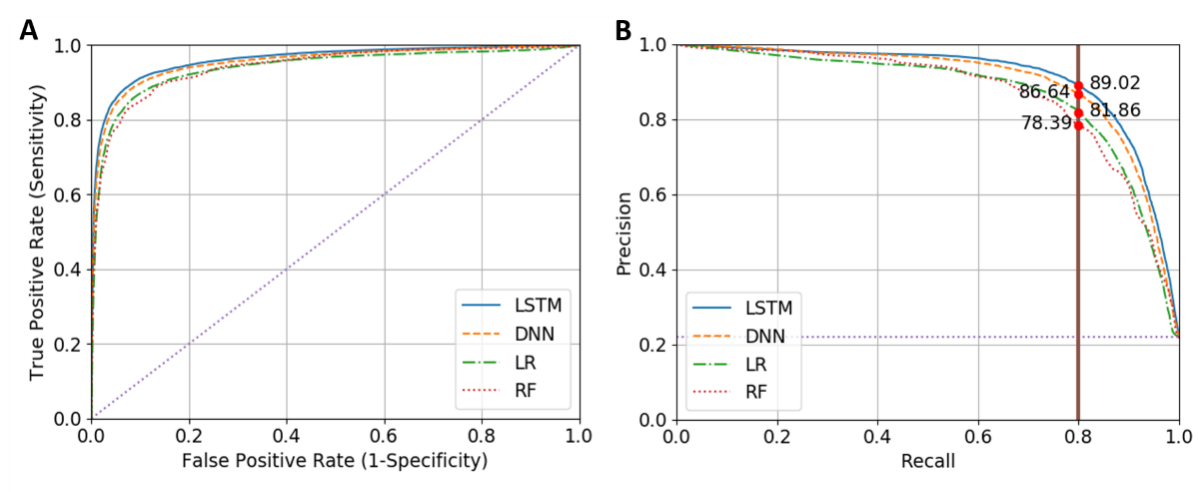
ROC and PR curves for all models. (A) ROC curve. Diagonal dotted purple line is the performance of random model. (B) PR curve. The vertical solid line shows precision of different models for achieving 0.8 recall. Straight dotted purple line is the performance of random model. DNN: dense neural network; LR: logistic regression; LSTM: long short-term memory; PR: precision-recall; RF: random forest; ROC: receiver operating-characteristic curve.

We compared our sequential-based model with other models based on a variety of metrics. As the data set is imbalanced (21.59% positive cases), accuracy cannot be a distinguishing metric among models. The area under the receiver operating characteristic curve (AUROC) also can be misleading in these data sets. The F1 score (harmonic mean of precision and recall) and area under the precision–recall curve (AUPRC) are more suitable metrics for this purpose [22,55,56]. In this project it is important to capture the majority of patients, therefore a model with high recall is desired. The precision for 0.80 recall is also measured and reported in Table 2. As shown in Figure 2, the LSTM-DNN model outperforms other models in both the AUROC and AUPRC curves. We excluded GRU-DNN in Figure 2 as it is close to the LSTM-DNN model.

**Table 2:** Methods performance comparison.

Models	Accuracy	Precision @0.8 recall	F1 score	AUPRC^a^	AUROC^b^
LSTM-DNN	93.04^c^	89.02^c^	84.30^c^	91.18^c^	96.15^c^
GRU-DNN	92.80	88.04	83.92	90.65	95.77
DNN	92.49	86.64	83.17	90.10	95.49
Logistic regression	90.77	81.86	80.03	86.47	93.96
Random forest	90.95	78.39	76.78	86.86	94.17

^a^AUPRC: area under the precision–recall curve.

^b^AUROC: area under the receiver operating characteristic curve.

^c^Numbers for the best method.

### Review Panel Validation Results

**Figure 3:**
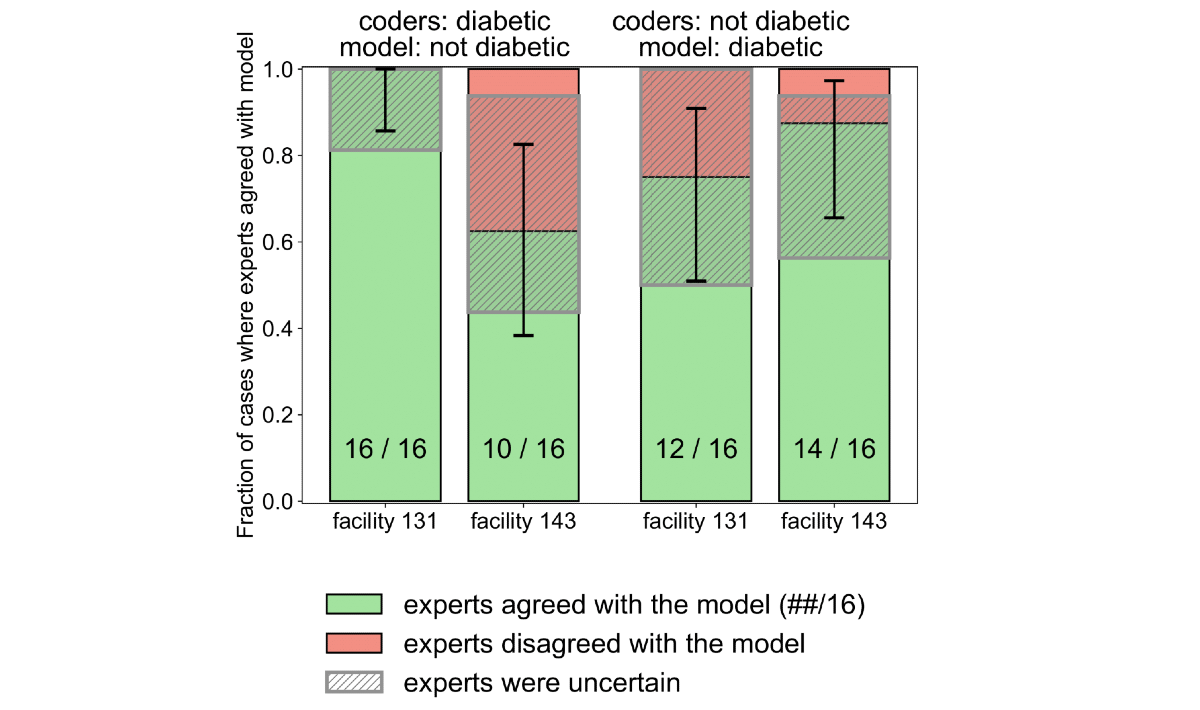
Expert review of cases where the model prediction disagreed with coded diagnosis. The error bars were 5% confidence intervals calculated from the beta binomial distribution.

For analyzing discordant cases where the model disagreed with what was recorded in the EHR, we performed a blinded review with a group of domain experts including at least three board-certified practicing physicians for each case review. For facilities 131 and 143, we used 32 sampled cases per facility where the model-predicted diagnosis was discordant with the EHR and the model had a high confidence (sigmoid output >0.83 or <0.17). We asked the review panel to answer 2 questions: (1) does the patient have diabetes; and (2) what is their confidence level? (high or low). In 52 out of the 64 cases, the panel’s conclusions agreed with the results from the model’s prediction. In 37 out of the 39 cases with which the panel had high confidence, the model’s prediction (output of the LSTM-DNN model) was consistent with the panel’s conclusion. Generally, the panel would have low confidence when there was insufficient evidence from the data to support a conclusion. The evaluation results are shown in Figure 3.

Through expert validation, we can provide a conservative estimate of how frequently a case flagged by the model for review would result in a correction at each facility. We calculated the range of the total population that would be potentially impacted for each facility with lower and upper bounds. The lower bound considers only the model’s high confidence interval—probability of more than 0.83 or less than 0.17 for positive and negative labeling, respectively, on sigmoid output—and the upper bound is for all predictions made by the model. Each value bound is multiplied by the probability of the model being correct, as derived from the expert validation (Figure 3). This final value is the percentage of the impacted population. In facility 131, we estimated that 1.25%-3.03% of the total population were missing a diabetes-related diagnosis code, and 1.65%-2.98% were improperly labeled as having diabetes. These numbers varied for facility 143, where there were 1.61%-3.73% missing a diabetes code and 1.12%-1.89% improperly labeled. Taking the mean of the intervals across facilities, we estimate that the error rate is 4.3% across these facilities. This suggests a considerable impact of this misclassification that can impact patients, hospitals, health systems, and payers.

These results demonstrate that when the model prediction contradicts the coders, the model is most often correct even for patients with several past encounters. From 32 cases with background information in 24 cases, experts agreed with the model. This suggests that a deep learning model trained from EHR data, which are often noisy, is capable of phenotyping and flagging cases for further review.

### Multiple Facilities Versus Single-Facility Models

In our study, we found that different facilities used different coding schemes for laboratory tests and medications. As a result, the diversity of features is higher than we had anticipated. For instance, blood glucose measurement, a standard test in diabetes, has a variety of names and Logical Observation Identifiers Names and Codes (LOINC) across facilities. Facilities reported “Glucose lab,” “Glucose [Mass/volume] in Blood,” “Glucose [Mass/volume] in Body fluid,” “Glucose [Mass/volume] in Blood by Test strip manual,” “Glucose; blood, reagent strip,” and “Glucose finger stick.” Each name has a different LOINC, making automated consolidation difficult. This problem exists in other data elements such as medications, where brand names, generic names, and various similar formulations are recorded. For this reason, a model trained on a single facility will not perform as well on another facility. Our goal was to develop a generalizable model that could perform well on all facilities independent of features available. Because features might vary widely, we proposed to collate all information from all facilities, and created 1 data set containing all features rather than manual or automatic merging of them (the data set we used for previous experiments). We were curious to see how does a model trained on this “combined” data set would differ from a model trained on just a single facility? From one perspective, with more data the model should perform better. However, as coding patterns and features vary significantly between facilities, this combination can end up misleading the model.

**Figure 4:**
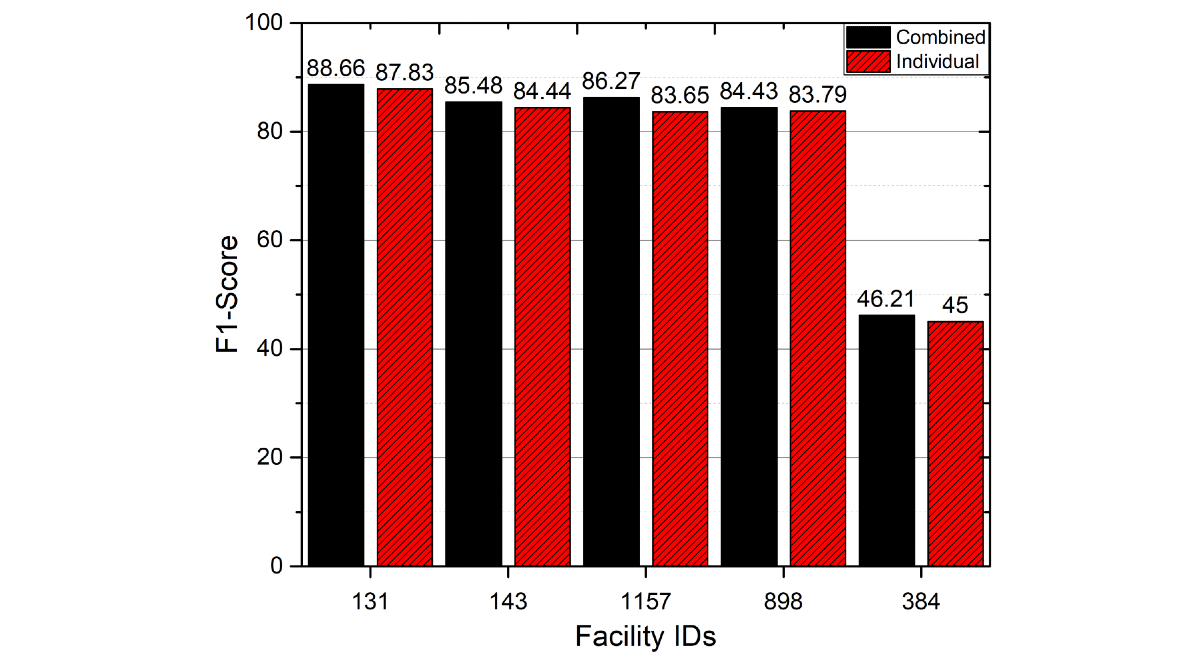
Comparison of F1 scores on single facility-based models and multifacility combined model.

We trained a model for each facility using the exact same steps we did previously using our best architecture (LSTM-DNN). As shown in Figure 4, the results from the combined model are very similar to those from the single facility–based models. In another experiment, we repeated the training on the combined data set without including facility IDs, and the results were almost the same. This suggests that the model trained on the combined data has the capability to learn all different patterns and can benefit from this approach.

Facility 384 showed very low performance, and we suspect that this is due to poor data quality and feature availability. We found that facility 384 reported fewer laboratory tests than other facilities (Table 1). It also lacked some laboratory tests essential to diabetes diagnosis, such as hemoglobin A1c. The facility also reported far fewer diagnoses per patient, including much lower prevalence of diabetes, even though it recorded metformin (a typical drug used for diabetes treatment) as much as other facilities. Thus, we believe that the low performance was due to the low availability of vital training features and the poor quality of recorded diagnosis codes. Interestingly, the model appeared to be resilient to other data problems, such as the paucity of medication data in facility 898.

### Limitations of Rule-Based Models

The traditional approach for phenotyping is based on a predefined set of rules and steps to determine whether a patient has a specific disease. To compare with such rule-based methods, we followed the steps in the eMERGE project [46]. Because of the lack of required data elements such as family history of diabetes and counts of dates that the patient had face-to-face outpatient clinic encounters, the performance of this algorithm was not ideal on our data set. For 75.28% of the patients, the results from the method were undecided and no final decision could be made. Another major limitation of such rule-based methods is the need for constant updates for new ICD codes, laboratory codes, and medications. Even after mapping and updating codes to current ICD-10, the method would often fail and detect only obvious cases and discard uncertain cases. As a result, it was not possible to make a reasonable comparison between models’ performances and the eMERGE criteria.

## Discussion

### Principal Findings

Our study demonstrates the successful identification of patient phenotypes using a deep learning model trained on heterogenous, minimally curated data. The model identifies a noticeable subset of potential coding errors in instances when patients are either improperly labeled as having or not having diabetes and is able to avoid errors arising from missing clinician documentation or sporadic coder errors. Given that the data were mapped to the OHDSI data model, the model is independent of facility-specific data representations and could be adopted by different health care systems based on normalization using OHDSI.

For much of the work on phenotyping, there is a presumption that the documented EHR diagnosis codes represent ground truth. However, human error can result in improper classification of a patient’s comorbidities and true illness severity. The motivation for this work was to detect and reclassify individuals in whom the wrong diagnosis was assigned at the time of discharge from the hospital, a fact that drives the development of such phenotyping algorithms. Our efforts can be used to flag discordant records for human review, leading to more accurate patient and population characterization. This strategy can be used to guide coders at the time of discharge to re-evaluate charts detected by the algorithm, with more directed attention to the potential missed diagnosis.

To validate the simulation of operational deployment of such a model, we used a double-blinded physician review panel to review the discordant cases where the model prediction was in contrary to the documented diagnosis. From this review, we not only captured the panel’s diagnosis but also the confidence level of their decision. During the review, the experts felt that some cases were too complex or needed more data for a model to classify correctly. Despite this, our panel and algorithm agreed on the final diagnosis among 81.25% of cases when the algorithm was confident in its prediction. In a real health system, this would equate to an anticipated 4 corrections to the coding for every 5 cases flagged by the model for further review. This is estimated to impact about 2.4% of a facility’s entire population missing a diabetes code that should be present, and about 1.9% of the population who were given the code of diabetes when it should not have been present. This suggests that our methodology is highly promising for improving clinical decision support to flag possibly missing or improper ICD classifications.

### Limitations

This work could benefit from expert validation at larger scale, which would result in a more accurate estimation of the effect on the population. As patients’ background information was very limited in this study, we did not expect significant difference using other methods such as attention-based models; however, they can be beneficial where more background data are available. Moreover, we are collaborating with the diabetes care group of our network hospitals to incorporate our prediction model into a pilot study.

### Conclusions

As research continues to advance the capabilities of predictive algorithms to medicine, we demonstrate a successful application of deep learning methodology bridging the gap at the intersection of computer science and clinical medicine.

We can classify a disease state in patients using a generalizable model that is deployable in institutions adopting the OHDSI standard. Our sequential deep learning–based model outperformed both traditional machine learning and nonsequential DNN as shown earlier. Results proved that the deep learning model can capture patterns for phenotyping from a high-dimension feature space without hand-crafted feature engineering. The findings also provide insights into how to build a framework/workflow using real-world EHR data for enhancing operations in real-world health care organizations, especially in applications to clinical intervention, documentation and billing, as well as quality improvement. The success of such disease prediction models can also benefit academic and translational research, as a faster and more refined disease phenotyping process allows researchers to better refine their study cohorts and minimize bias or confounding variables. Most importantly, one cannot understate the potential impact to patient care and clinical outcomes afforded by this approach to diagnostic validation and case ascertainment.

## Appendix

Multimedia Appendix 1 Population demographic statistics.

Multimedia Appendix 2 Excluded ICD-10 codes from CCS 49, 50, 186.

## Abbreviations

AUPRC: area under the precision–recall curve
AUROC: area under receiver operating-characteristic curve
CCS: Clinical Classification Software
DNN: dense neural network
EHR: electronic health record
GRU: gated recurrent unit
LOINC: Logical Observation Identifiers Names and Codes
LSTM: long short-term memory
OHDSI: Observational Health Data Sciences and Informatics
RNN: recurrent neural network
