# Supplementary material for: Detecting Miscoded Diabetes Diagnosis Codes in Electronic Health Records for Quality Improvement: Temporal Deep Learning Approach
Source: JMIR Med Inform. 2020 Dec 17;8(12):e22649. doi: 10.2196/22649 (PMC7775195; doi:10.2196/22649)
Supplement: Multimedia Appendix 1 [file medinform_v8i12e22649_app1.docx]

**Population demographic statistics**

We extracted data for adults for the 5 acute care facilities with the most inpatient discharges during 1/1/2016 - 12/31/2017 from Cerner Healthfacts database.

| **Demographic Info** | **Absolute Count** | **Relative Count (%)** |
| --- | --- | --- |
| Age group (18-28) | 21656 | 11.56 |
| Age group (29-38) | 31068 | 16.59 |
| Age group (39-48) | 21221 | 11.33 |
| Age group (49-58) | 29124 | 15.55 |
| Age group (59-68) | 32403 | 17.31 |
| Age group (69-78) | 25677 | 13.71 |
| Age group (+79) | 26038 | 13.87 |
| Male | 82844 | 44.25 |
| Female | 104333 | 55.73 |
| White | 98042 | 52.37 |
| African American | 42377 | 22.63 |
| Asian | 4281 | 2.28 |
