# Supplementary material for: Detecting Miscoded Diabetes Diagnosis Codes in Electronic Health Records for Quality Improvement: Temporal Deep Learning Approach
Source: JMIR Med Inform. 2020 Dec 17;8(12):e22649. doi: 10.2196/22649 (PMC7775195; doi:10.2196/22649)
Supplement: Multimedia Appendix 2 [file medinform_v8i12e22649_app2.docx]

**Excluded ICD-10 codes from CCS 49, 50, 186**

**CCS 49 “Diabetes mellitus without complications” (7 codes)**

- R73.01 Impaired fasting glucose
- R73.02 Impaired glucose tolerance (oral)
- R73.03 Prediabetes
- R73.09 Other abnormal glucose
- R73.9 Hyperglycemia, unspecified
- R81 Glycosuria
- R82.4 Acetonuria

**CCS 50 “Diabetes mellitus with complications” (0 codes)**

**CCS 186 “Complications mainly related to pregnancy” (22 codes)**

- O24.410 Gestational diabetes mellitus in pregnancy, diet controlled
- O24.414 Gestational diabetes in pregnancy, insulin controlled
- O24.415 Gestational diabetes mellitus in pregnancy, controlled by oral hypoglycemic drugs
- O24.419 Gestational diabetes mellitus in pregnancy, unsp control
- O24.420 Gestational diabetes mellitus in childbirth, diet controlled
- O24.424 Gestational diabetes in childbirth, insulin controlled
- O24.425 Gestational diabetes mellitus in childbirth, controlled by oral hypoglycemic drugs
- O24.429 Gestational diabetes mellitus in childbirth, unsp control
- O24.430 Gestational diabetes in the puerperium, diet controlled
- O24.434 Gestational diabetes in the puerperium, insulin controlled
- O24.435 Gestational diabetes mellitus in puerperium, controlled by oral hypoglycemic drugs
- O24.439 Gestational diabetes in the puerperium, unsp control
- O24.911 Unspecified diabetes mellitus in pregnancy, first trimester
- O24.912 Unspecified diabetes mellitus in pregnancy, second trimester
- O24.913 Unspecified diabetes mellitus in pregnancy, third trimester
- O24.919 Unspecified diabetes mellitus in pregnancy, unspecified trimester
- O24.92 Unspecified diabetes mellitus in childbirth
- O24.93 Unspecified diabetes mellitus in the puerperium
- O99.810 Abnormal glucose complicating pregnancy
- O99.814 Abnormal glucose complicating childbirth
- O99.815 Abnormal glucose complicating the puerperium
